# Orchestrated Action of PP2A Antagonizes Atg13 Phosphorylation and Promotes Autophagy after the Inactivation of TORC1

**DOI:** 10.1371/journal.pone.0166636

**Published:** 2016-12-14

**Authors:** Akter MST Yeasmin, Talukdar Muhammad Waliullah, Akihiro Kondo, Atsuki Kaneko, Naoki Koike, Takashi Ushimaru

**Affiliations:** 1 Graduate School of Science and Technology, Shizuoka University, Suruga-ku, Shizuoka, Japan; 2 Faculty of Science, Shizuoka University, Suruga-ku, Shizuoka, Japan; Univerzitet u Beogradu, SERBIA

## Abstract

Target of rapamycin complex 1 (TORC1) phosphorylates autophagy-related Atg13 and represses autophagy under nutrient-rich conditions. However, when TORC1 becomes inactive upon nutrient depletion or treatment with the TORC1 inhibitor rapamycin, Atg13 dephosphorylation occurs rapidly, and autophagy is induced. At present, the phosphatases involved in Atg13 dephosphorylation remain unknown. Here, we show that two protein phosphatase 2A (PP2A) phosphatases, PP2A-Cdc55 and PP2A-Rts1, which are activated by inactivation of TORC1, are required for sufficient Atg13 dephosphorylation and autophagy induction after TORC1 inactivation in budding yeast. After rapamycin treatment, dephosphorylation of Atg13, activation of Atg1 kinase, pre-autophagosomal structure (PAS) formation and autophagy induction are all impaired in PP2A-deleted cells. Conversely, overexpression of non-phosphorylatable Atg13 suppressed defects in autophagy in PP2A mutant. This study revealed that the orchestrated action of PP2A antagonizes Atg13 phosphorylation and promotes autophagy after the inactivation of TORC1.

## Introduction

Autophagy (or macroautophagy) degrades cytoplasmic components in lysosomes/vacuoles, which is a conserved system from the yeast to mammalian cells [1, 2]. Newly generated cup-shaped structures, called isolation membranes, expand to encapsulate cellular cargos and then the edges of isolation membranes fuse to form double membrane-surrounded autophagosomes. Subsequently, autophagosomes fuse with lysosomes/vacuoles, and the engulfed cargoes are digested by lysosomal hydrolytic enzymes.

In response to nutrient starvation, autophagy is dramatically induced to recycle proteins and other cellular components. Target of rapamycin complex 1 (TORC1), a nutrient-responsive protein kinase, regulates autophagy induction [3]. TORC1 phosphorylates Atg13 under nutrient-sufficient conditions, but TORC1 is inactivated under nutrient-starved conditions or by the specific TORC1 inhibitor rapamycin, which causes Atg13 dephosphorylation [4]. Dephosphorylated Atg13 forms a complex, called the Atg1 kinase complex, consisting of Atg1 kinase, Atg13, Atg17, Atg29 and Atg31 [4–6]. Formation of the Atg1 complex is required for Atg1 kinase activation and for the initial step of autophagosome formation, although the critical targets of Atg1 remain elusive. Expression of a non-phosphorylatable Atg13 mutant partially induced Atg1 complex formation and autophagy in non-starved cells, indicating that dephosphorylation of Atg13 is sufficient for autophagy induction [7]. The TORC1-Atg1 signaling axis is conserved throughout most eukaryotes to regulate autophagy [1, 2]. In addition to TORC1, protein kinase A is involved in Atg13 phosphorylation [8]. Thus, some protein kinases involved in autophagy repression and Atg13 phosphorylation have been identified, whereas the protein phosphatases responsible for autophagy induction and Atg13 dephosphorylation remain unknown.

Protein phosphatase 2A (PP2A) is functionally and structurally conserved from yeast to human and plays a crucial role in regulating various cellular events, including development and cell proliferation and death [9–11]. TORC1 negatively regulates protein phosphatase 2A (PP2A; Pph21 and Pph22), and PP2A-related protein phosphatases 4 (PP4, Pph3) and 6 (PP6; Sit4) in the budding yeast *Saccharomyces cerevisiae* via Tap42 [9]. PP2A consists of three subunits, namely, the catalytic C subunit (Pph21 and Pph22), the scaffold A subunit (Tpd3) and the regulatory B subunit (Cdc55 and Rts1) in yeast [9]. Pph21/Pph22 associated with Cdc55 (PP2A-Cdc55) and PP2A-associated with Rts1 (PP2A-Rts1) have distinct functions and substrate specificity. TORC1 negatively regulates both PP2A-Cdc55 and PP2A-Rts1 via Tap42: TORC1 promotes phosphorylation of Tap42, and phosphorylated Tap42 binds to PP2A, competitively with Tpd3, Cdc55 and Rts1, repressing formation of PP2A-Cdc55 and PP2A-Rts1 [12]. Upon TORC1 inactivation, Tap42 is dephosphorylated, which promotes formation of PP2A-Cdc55 and PP2A-Rts1. PP2A-Cdc55 mediates Tap42 dephosphorylation as a positive feedback loop [12]. In addition, PP2A-Cdc55 acts as an antagonist of TORC1 signaling; PP2A-Cdc55 mediates dephosphorylation of TORC1 downstream proteins after the inactivation of TORC1 [13]. In addition, PP2A-Rts1 shares a redundant function with PP2A-Cdc55 after the inactivation of TORC1 [14].

Here, we show that PP2A (PP2A-Cdc55 and PP2A-Rts1) is required for autophagy induction after the inactivation of TORC1. Furthermore, we demonstrate that PP2A is involved in Atg13 dephosphorylation, Atg1 complex formation, Atg1 activation, and pre-autophagosomal structure (PAS) formation.

## Materials and Methods

### Strains, plasmids, and media

*S*. *cerevisiae* strains and plasmids used are listed in S1 and S2 Tables, respectively. Glucose-containing YPAD (YPD containing 0.01% adenine) and synthetic minimal medium (SD) complemented with the appropriate nutrients for plasmid maintenance were prepared using standard methods. For raffinose-based media, 2% raffinose plus 3% glycerol were used instead of 2% glucose. For assessment of autophagy, when cells harbor plasmids, cells were precultured in SD with the appropriate nutrients, and then cultured in YPAD. For nitrogen-starvation experiments, cells were transferred into SD-N without ammonium sulfate. Rapamycin was added to the medium to a final concentration of 0.2 μg/ml. Rapamycin was diluted into media from a stock solution of 50 μg/ml in 10% Tween-20/90% ethanol.

### Western blotting analysis

Proteins were extracted by a post-alkaline extraction method in accordance with a previous report [15]. Briefly, cells (10 ml culture, OD_600_ = 0.2–0.8) were treated with 200 μl of 0.1 M NaOH for 5 min and then the pellet was collected by centrifugation. The pellet was resuspended in sample buffer (60 mM Tris-HCl (pH 6.8), 5% glycerol, 2% SDS, 4% 2-mercaptoethanol and 0.0025% bromophenol blue) at 95°C for 5 min. Crude extracts were cleared by centrifugation and the supernatant was used for western blotting analysis. We used the following antibodies: anti-GFP mouse monoclonal antibody (Santa Cruz, #sc-9996), anti-Ape1 rabbit polyclonal antibody (a gift from D. Klionsky), anti-Atg1 rabbit polyclonal antibody (a gift from Y. Ohsumi), anti-Atg13 rabbit polyclonal antibody (a gift from Y. Kamada), an anti-CDK rabbit polyclonal antibody (Santa Cruz, #sc-53) and an anti-Pgk1 mouse monoclonal antibody (Thermo Fisher Scientific, #A-6457). Chemiluminescence signals from Western BLoT Quant HRP Substrate (Takara, #DS-T7102) for horseradish peroxidase (HRP) and Immuno Shot (Cosmo Bio, #IS-012-250) as an immunoreaction enhancer solution were detected using an image analyser (Fuji LAS3000mini). For detection of phosphorylation statuses of Atg13 and Atg1, 7.5% acrylamide gels were used for SDS-PAGE. All western blotting experiments were performed at least twice independently to confirm reproducibility of the results. Relative protein amounts were measured using ImageJ software. The average was determined for each sample of two independent experiments and relative values normalized against the value in control cells are shown.

### Microscope observations

Cell, GFP and RFP images were captured using a Carl Zeiss Axio Imager M1 microscope with a cooled CCD camera (Carl Zeiss AxioCam MRm). For examination of PAS formation, more than 100 cells with Atg8- or Atg1-marked puncta were counted and were scored. All microscope observations were performed at least twice independently to confirm reproducibility of the results. Data are shown as means ± errors. Statistical analyses were carried out using Fisher's exact test. Statistical analyses were performed at least twice independently to confirm reproducibility of the results.

### Rosella assay

We used cells expressing cytoplasmic “Rosella” consisting to pH-sensitive GFP fused to RFP [16]. We measured the fluorescence intensities of GFP and RFP in the cytoplasm and vacuole using a microscope and imaging software. Two GFP fluorescence peaks in cytoplasmic regions and lower signals in the vacuole were detected in cells without autophagy (S2A Fig, Control). Here, we defined the values of the lower peak of the two peaks in the cytoplasm and of the bottom in the vacuole as those of cytoplasmic GFP (GFPc) and vacuolar GFP (GFPv), respectively (S2B Fig). Intensity profiles of GFP-fused RFP were very similar to those of GFP (S2A Fig, Control, Merge), and we defined values of cytoplasmic (RFPc) and vacuolar (RFPv) RFP in the same way.

**Fig 1:**
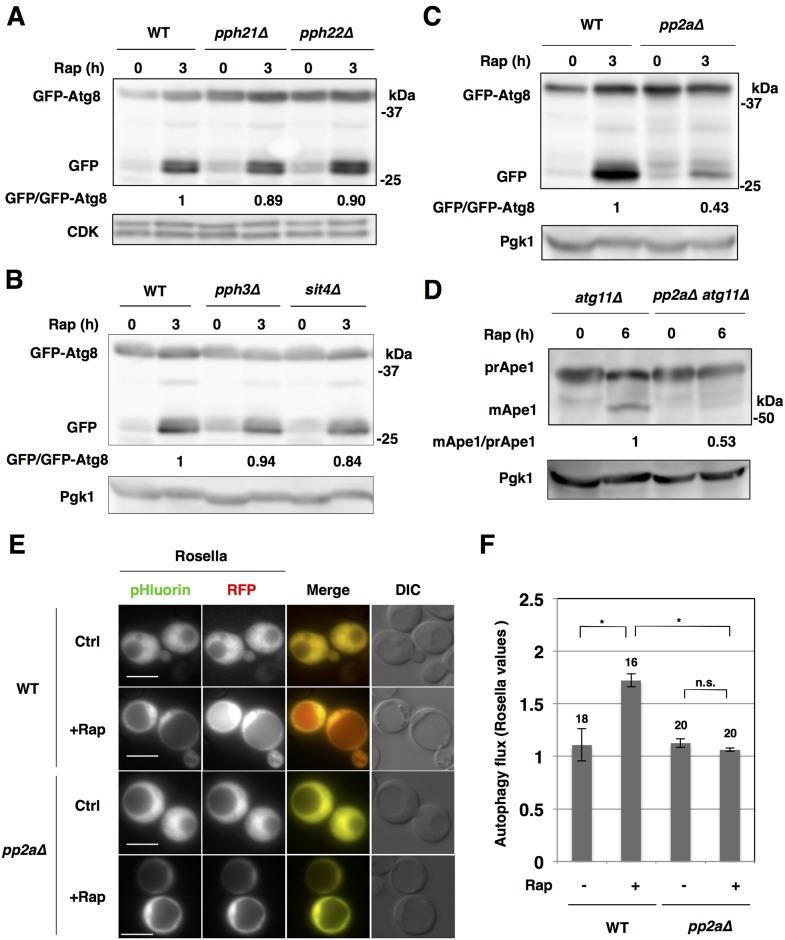
PP2A is required for autophagy induction after the inactivation of TORC1. (A) Exponentially growing cells of strains BY4741 (wild type; WT), SCU3086 (*pph21*Δ) and SCU3087 (*pph22*Δ) harboring plasmid pSCU1998 (pGFP-ATG8) were treated with 200 ng/ml rapamycin for 3 h. Whole cell extracts were subjected to western blotting using an anti-GFP antibody. Cyclin-dependent kinase (CDK) was detected as the loading control using an anti-CDK antibody. All western blotting experiments were performed at least twice independently to confirm reproducibility of the results. Free GFP processed from GFP-tagged protein was measured using ImageJ software, and quantified by calculating the ratio of cleaved free GFP versus uncleaved full-length protein. The average was determined for each sample of two independent experiments and relative values normalized against the value in control cells are shown. (B) Cells of strains BY4741 (wild type; WT), SCU3088 (*pph3*Δ) and SCU2142 (*sit4*Δ) harboring plasmid pSCU1998 (pGFP-ATG8) were treated with rapamycin for 3 h. Whole cell extracts were subjected to western blotting. Pgk1 was detected as the loading control using an anti-Pgk1 antibody. (C) Cells of strains SCU893 (wild type, isogenic to W303) and SCU2422 (*pph21*Δ *pph22*Δ; *pp2a*Δ) harboring a plasmid pSCU1998 were treated with rapamycin for 3 h. Whole cell extracts were subjected to western blotting using an anti-GFP antibody. (D) Ape1 processing assays indicated that autophagy induction after rapamycin treatment is repressed in *pp2a*Δ cells. Cells of strains SCU3720 (*atg11*Δ) and SCU3736 (*pph21*Δ *pph22*Δ *atg11*Δ) were treated with rapamycin for 6 h. Whole cell extracts were subjected to western blotting and pre-Ape1 (preApe1) and mature Ape1 (mApe1) were detected using an anti-Ape1 antibody. Mature Ape1 processed from pre-Ape1 was measured using ImageJ software, and quantified by calculating the ratio of mature Ape1 versus pre-Ape1. Relative values normalized against the value in control cells are shown. (E, F) Cells of strains SCU893 (wild-type) and SCU2422 (*pph21*Δ *pph22*Δ) harboring a plasmid pSCU2260 expressing Rosella (pH-sensitive GFP fused to RFP) were treated with rapamycin for 18 h (+Rap). Rapamycin-untreated cells were used as the control (Ctrl). Representative images of cells are shown (E). Scale bars, 5 μm. GFP and RFP signals in the cytoplasm and vacuole were quantified in cells and the Rosella values (see “Materials and Methods”) were calculated and shown along with statistical analysis in (F). Numbers above the bars are sample sizes. The error bars indicate SEM. n.s., non-significant; *, P < 0.01 (Fisher’s exact test).

When Rosella was imported into the vacuole by nonselective autophagy, signals of RFP, but not GFP, were increased in the vacuole. Quantification analysis indicated that the RFP signal was higher in the vacuole than in the cytoplasm, and we defined the highest value in the vacuole as the value of RFPv (S2B Fig, WT+Rap). In this case, we defined a value of the RFP signal at the scanning position where GFPc was determined, as the value of RFPc. We calculated the value of {(RFPv-base)/(RFPc-base)}/{(GFPv-base)/(GFPc-base)} in each cell and defined this value as the Rosella value (index of autophagy flux). When autophagy did not occur, this value was 1; when autophagy was induced, the value was more than 1. Microscope observations were performed at least twice independently to confirm reproducibility of the results. Data are shown as means ± SEM. Statistical analyses were carried out using Fisher's exact test in Fig 1F.

## Results

### PP2A is required for autophagy induction after the inactivation of TORC1

We investigated which protein phosphatases are involved in autophagy induction via inactivation of TORC1. First, we suspected that PP2A (Pph21 and Pph22), PP4 (Pph3) and/or PP6 (Sit4) might be involved in autophagy induction upon inactivation of TORC1, because these are negatively regulated by TORC1 [9]. We monitored autophagy induction after rapamycin treatment (monitored by GFP-Atg8 cleavage; [17]) in single mutants, *pph21*Δ, *pph22*Δ, *sit4*Δ and *pph3*Δ. However, we found no significant alterations in autophagy induction in these mutant cells (Fig 1A and 1B). This suggested that PP4 and PP6 are not critically implicated in autophagy induction after the inactivation of TORC1.

Therefore, we focused on the homologous PP2A phosphatases Pph21 and Pph22, which have redundant functions. Autophagy induction (monitored by GFP-Atg8 processing) after rapamycin treatment was impaired in *pph21*Δ *pph22*Δ (*pp2a*Δ, herein) cells (Fig 1C). This indicated that rapamycin-induced autophagy was compromised in *pp2a*Δ cells.

TORC1 inactivation induces nonselective autophagy. Atg11 is a receptor protein for selective autophagy, including the constitutive autophagy-like system, the biosynthetic cytoplasm to vacuole targeting (Cvt) pathway, mitophagy and pexophagy [18]. Ape1 processing assay in *atg11*Δ background, which is used for assessment for nonselective autophagy flux [19], indicated that induction of nonselective autophagy after TORC1 inactivation was compromised in *pp2a*Δ cells (Fig 1D). Consistently, the GFP-Atg8 processing assay in an *atg11*Δ background (an index of nonselective autophagy flux) also showed nonselective autophagy defects in *pp2a*Δ cells (S1A Fig). In contrast, Ape1 maturation via the constitutive Cvt pathway under nutrient-rich conditions [20] was not compromised in *pp2a*Δ cells in *ATG11* background (S1B Fig). This indicates that PP2A is dispensable for the Cvt pathway.

In addition, we utilized a “Rosella” biosensor for nonselective autophagy assessment [16]. Rosella consists of a pH-sensitive GFP (pHluorin) fused to red fluorescent protein (RFP), which is expressed in the cytoplasm. In normal (nutrient-rich) conditions, the green and red signals in the cytoplasm were higher than in the vacuole (Fig 1E and S2A Fig, WT, Ctrl). Both signals overlapped in the cytoplasm and vacuole. After rapamycin treatment, Rosella was transferred to the vacuole by nonselective autophagy, resulting in the red signal becoming stronger in the vacuole than in the cytoplasm, whereas the green signal was still weaker in the vacuole than in the cytoplasm, because it was lost in the vacuole (Fig 1E and S2A Fig, WT+Rap). However, such increases in the red signal in the vacuole after rapamycin treatment were not detected in *ppa2*Δ cells (Fig 1E and S2A Fig, *pp2a*Δ+Rap). The Rosella assay and its quantification analysis again indicated that nonselective autophagy induction after TORC1 inactivation was remarkably repressed in *pp2a*Δ cells (Fig 1E, 1F, and S2A, S2B Fig). Thus, PP2A is required for induction of nonselective autophagy after TORC1 inactivation.

### Both PP2A-Cdc55 and PP2A-Rts1 are involved in autophagy induction

**Fig 2:**
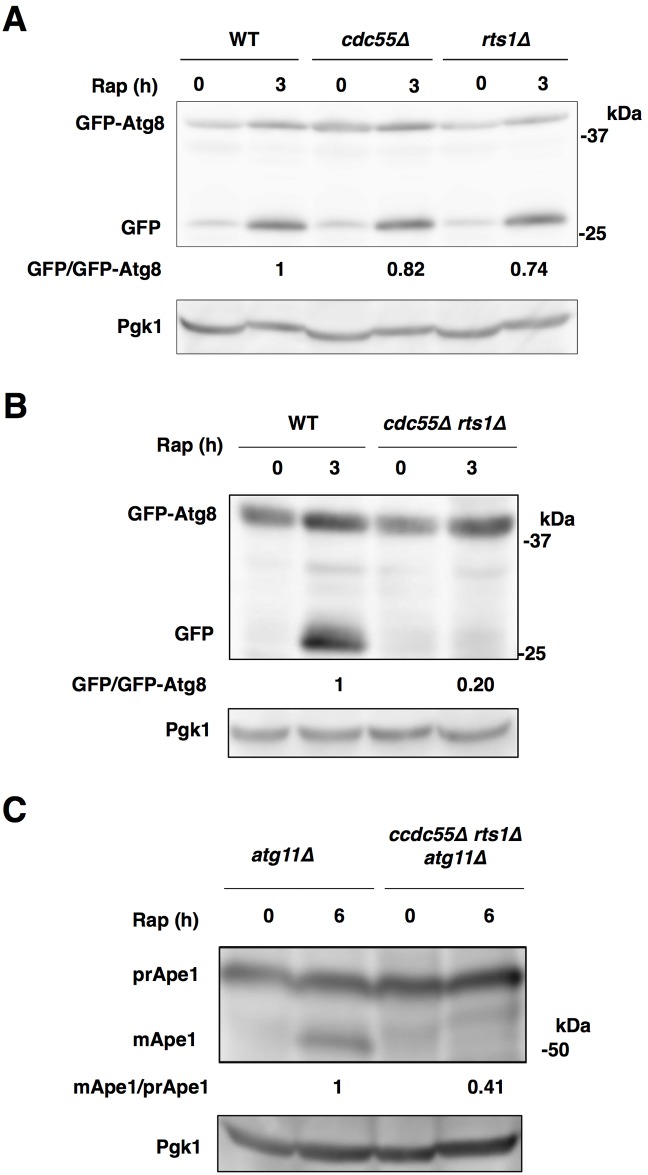
Both PP2A-Cdc55 and PP2A-Rts1 are involved in autophagy induction. (A) Cells of strains SCU893 (wild-type), SCU4221 (*cdc55*Δ) and SCU4223 (*rts1*Δ) harboring plasmid pSCU1998 (pGFP-ATG8) were treated with rapamycin for 3 h. (B) Cells of strains SCU893 (wild-type) and SCU4225 (*cdc55*Δ *rts1*Δ) harboring plasmid pSCU1998 were treated with rapamycin for 3 h. Whole cell extracts were subjected to western blotting using the anti-GFP antibody. (C) Cells of strains SCU3720 (*atg11*Δ) and SCU4069 (*cdc55*Δ *rts1*Δ *atg11*Δ) were treated with rapamycin for 6 h. Whole cell extracts were subjected to western blotting using the anti-Ape1 antibody.

PP2A forms two distinct complexes, PP2A-Cdc55 and PP2A-Rts1, which are both negatively regulated by TORC1 [9]. After the inactivation of TORC1, PP2A-Cdc55 mediates dephosphorylation of TORC1 downstream proteins and PP2A-Rts1 also shares a redundant function with PP2A-Cdc55 [13, 14]. Autophagy induction (monitored by GFP-Atg8 processing) after rapamycin treatment was not remarkably suppressed in either *cdc55*Δ or *rts1*Δ single mutant cells (Fig 2A), unlike *pp2a*Δ cells (Fig 1C). Therefore, we created *cdc55*Δ *rts1*Δ double mutant cells. PP2A activity is not essential for viability, but *pp2a*Δ cells showed a slow cell growth phenotype (S3A Fig). In contrast, the cell growth defect was less pronounced in *cdc55*Δ *rts1*Δ cells than in *pp2a*Δ. This suggested that PP2A activity was not completely abolished in *cdc55*Δ *rts1*Δ cells. However, autophagy induction was again repressed in *cdc55*Δ *rts1*Δ cells (Fig 2B), like *pp2a*Δ cells (Fig 1C).

In addition, rapamycin-induced nonselective autophagy was also repressed in *cdc55*Δ *rts1*Δ cells (monitored by Ape1 and GFP-Atg8 processing in *atg11*Δ background; Fig 2C and S3B Fig). These indicated that PP2A-Cdc55 and PP2A-Rts1 have a redundant function in induction of TORC1 inactivation-induced (nonselective) autophagy. In contrast, the Cvt pathway was not impeded in *cdc55*Δ *rts1*Δ cells, like in *pp2a*Δ cells (S1B Fig), supporting that PP2A is not critical for the Cvt pathway.

### PP2A is required for autophagy induction after nitrogen starvation

Nitrogen starvation causes the inactivation of TORC1 and is a natural trigger of autophagy induction. However, in addition to TORC1, there are many protein kinases that participate in autophagy regulation, including cyclin-dependent kinases (CDKs), Cdc28 and Pho85, the Ras/cAMP-dependent protein kinase A (PKA), Sch9, a homolog of mammalian protein kinase B (PKB)/Akt, amino acid-responsive Gcn2, Greatwall kinase Rim15, which is required for the establishment of the G0 program in response to nutrients, and Ksp1, which is involved in the TOR signaling cascade [21–27]. How these protein kinases respond to nutrient starvation and rapamycin treatment to regulate autophagy remains elusive. It is likely that responses of these other protein kinases to nitrogen starvation might be different from those to rapamycin treatment. Therefore, different sets of protein phosphatases could participate in autophagy regulation after rapamycin treatment and nitrogen depletion. We tested whether PP2A is also critical for autophagy induction after nitrogen starvation. GFP-Atg8 processing and Ape1 processing in the *atg11*Δ background after nitrogen starvation were both repressed in *pp2a*Δ cells (S4A and S4B Fig). Similarly, nitrogen starvation-induced autophagy was also compromised in *cdc55*Δ *rts1*Δ cells (S4C and S4D Fig). Thus, PP2A is also critical for nitrogen depletion-induced autophagy (nonselective autophagy).

### PP2A is required for sufficient Atg13 dephosphorylation after the inactivation of TORC1

**Fig 3:**
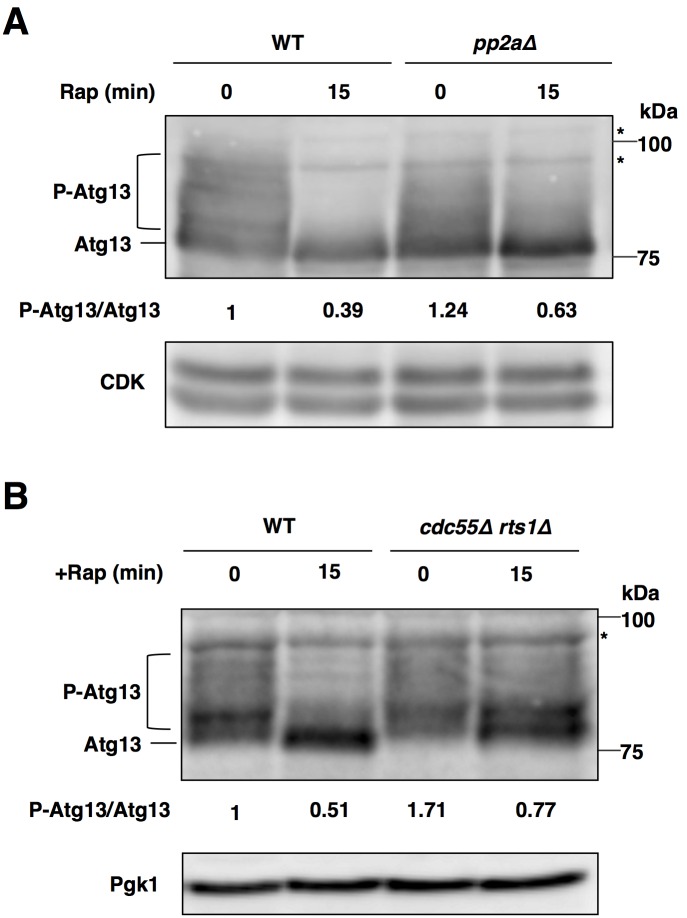
PP2A is required for sufficient Atg13 dephosphorylation after the inactivation of TORC1. (A) Cells of strains SCU893 (wild type, isogenic to W303a) and SCU2422 (*pph21*Δ *pph22*Δ) harboring plasmid pSCU1984 (pATG13) were treated with rapamycin for 15 min. Whole cell extracts were subjected to western blotting using an anti-Atg13 antibody. For detection of phosphorylation statuses of Atg13, 7.5% acrylamide gels were used. Phosphorylated Atg13 was measured using ImageJ software, and quantified by calculating the ratio of phosphorylated Atg13 versus dephosphorylated Atg13. Relative values normalized against the value in control cells are shown. P-Atg13, phosphorylated Atg13. Asterisks depict non-specific bands. (B) Cells of strains SCU893 (wild type) and SCU4225 (*cdc55*Δ *rts1*Δ) harboring plasmid pSCU1984 (pATG13) were treated with rapamycin for 15 min. Whole cell extracts were subjected to western blotting as for panel (A).

Dephosphorylation of Atg13 is the key event in triggering the induction of autophagy after the inactivation of TORC1 [1, 28]. We suspected that the defect in autophagy induction after the inactivation of TORC1 in *pp2a*Δ cells might be due to repression of Atg13 dephosphorylation. Atg13 is hyperphosphorylated under favorable nutrient conditions and dephosphorylated after rapamycin treatment in wild-type cells (Fig 3A). However, the dephosphorylation of Atg13 upon rapamycin treatment was partially impeded in *pp2a*Δ cells. In addition, Atg13 dephosphorylation after rapamycin treatment was partially impaired in *cdc55*Δ *rts1*Δ cells (Fig 3B). These indicated that PP2A is required for sufficient Atg13 dephosphorylation after the inactivation of TORC1. In normal conditions, the ratio of phosphorylated Atg13 versus dephosphorylated Atg13 was higher in *pp2a*Δ cells than in wild-type cells, although a dephosphorylated form of Atg13 appeared to be accumulated in *pp2a*Δ cells compared with the wild-type cells (Fig 3A). Similarly, phosphorylated forms of Atg13 were more accumulated in *cdc55*Δ *rts1*Δ cells than in wild-type cells in normal conditions (Fig 3B). Thus, PP2A was also involved in dephosphorylation of Atg13 in TORC1 active conditions.

### PP2A is required for sufficient Atg1 activation after the inactivation of TORC1

**Fig 4:**
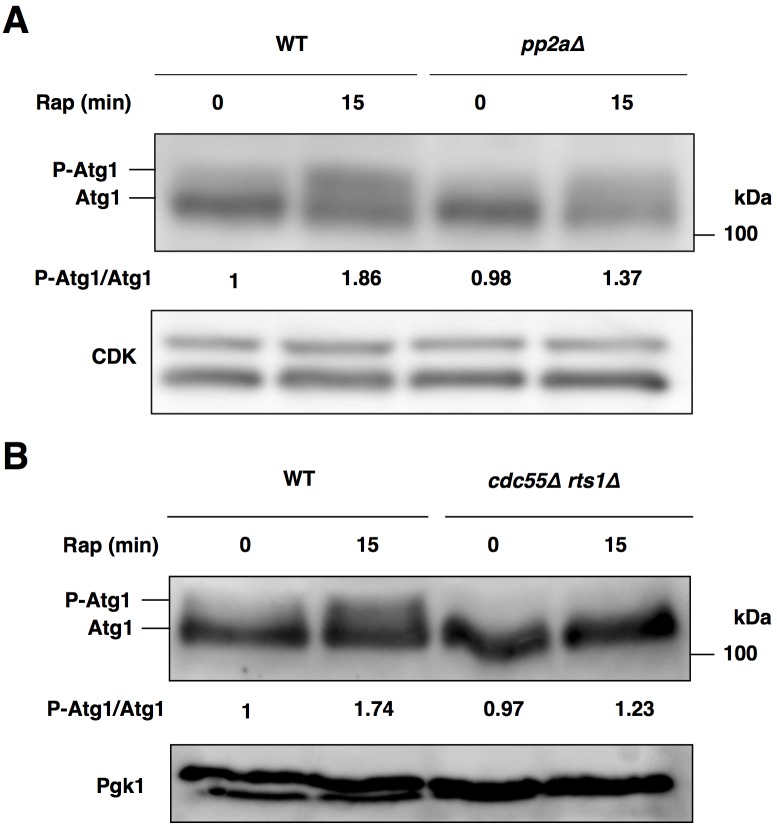
PP2A is required for Atg1 activation after the inactivation of TORC1. (A) Cells of strains SCU893 (wild type) and SCU2422 (*pph21*Δ *pph22*Δ) were treated with rapamycin for 15 min. Whole cell extracts were subjected to western blotting using an anti-Atg1 antibody. Phosphorylated Atg1 was measured using ImageJ software, and quantified by calculating the ratio of phosphorylated Atg1 versus dephosphorylated Atg1. Relative values normalized against the value in control cells are shown. P-Atg1, phosphorylated Atg1. (B) Cells of strains SCU893 (wild type) and SCU4225 (*cdc55Δ rts1*Δ) were treated with rapamycin for 15 min. Whole cell extracts were subjected to western blotting as for panel (A).

After rapamycin treatment, interaction of dephosphorylation of Atg13 to Atg1 is required for Atg1 kinase activation and autophagy induction [5]. Therefore, the reduced protein levels of Atg13 and insufficient dephosphorylation of Atg13 in *pp2a*Δ cells after rapamycin treatment may compromise Atg1 activation. Activated Atg1 can be monitored as a slower-migrating band in SDS-PAGE gels, owing to autophosphorylation [29]. The slower-migrating band (activated Atg1) became prominent in wild-type cells after rapamycin treatment, whereas the appearance of activated Atg1 after rapamycin treatment was partially repressed in *pp2a*Δ and *cdc55*Δ *rts1*Δ cells (Fig 4A and 4B). Thus, PP2A is required for the full activation of Atg1 after the inactivation of TORC1. In sum, PP2A-Cdc55 and PP2A-Rts1 are required for sufficient Atg13 dephosphorylation and Atg1 kinase activation.

### PP2A is required for Ape1-independent PAS formation after TORC1 inactivation

The PAS proximal to the vacuole is a putative site for autophagosome formation in yeast [30, 31]. The PAS might generate an isolation membrane, which is the precursor of the autophagosome. Most Atg proteins, including Atg8 and Atg1 are localized to the PAS under starvation conditions, which is prerequisite for formation of the autophagosome [6, 32, 33]. Atg8 is a key Atg protein for elongation of the isolation membrane and formation of the autophagosome [2]. On the other hand, the PAS is also organized for the Cvt pathway under nutrient-rich conditions. Atg8 was recruited to the PAS in *pp2a*Δ cells under such conditions, to a similar extent as that found in wild-type cells (S5A and S5B Fig). However, prominent punctate signals were increased after rapamycin treatment in the wild-type cells. The puncta were located in a perivacuolar region and represent the phagophore assembly site (S5A and S5B Fig, Merge in WT+Rap). Perivacuolar localization of the puncta was further confirmed using the vacuolar membrane dye FM4-64 (data not shown). This PAS localization of Atg8 after rapamycin treatment was largely compromised in *pp2a*Δ cells. Similarly, PAS formation was compromised in *cdc55Δ rts1*Δ double mutant cells (S6A and S6B Fig). In addition, PAS localization of Atg1 after rapamycin treatment was also repressed in *pp2a*Δ cells (S5C and S5D Fig). Thus, PP2A promotes PAS assembly after the inactivation of TORC1.

**Fig 5:**
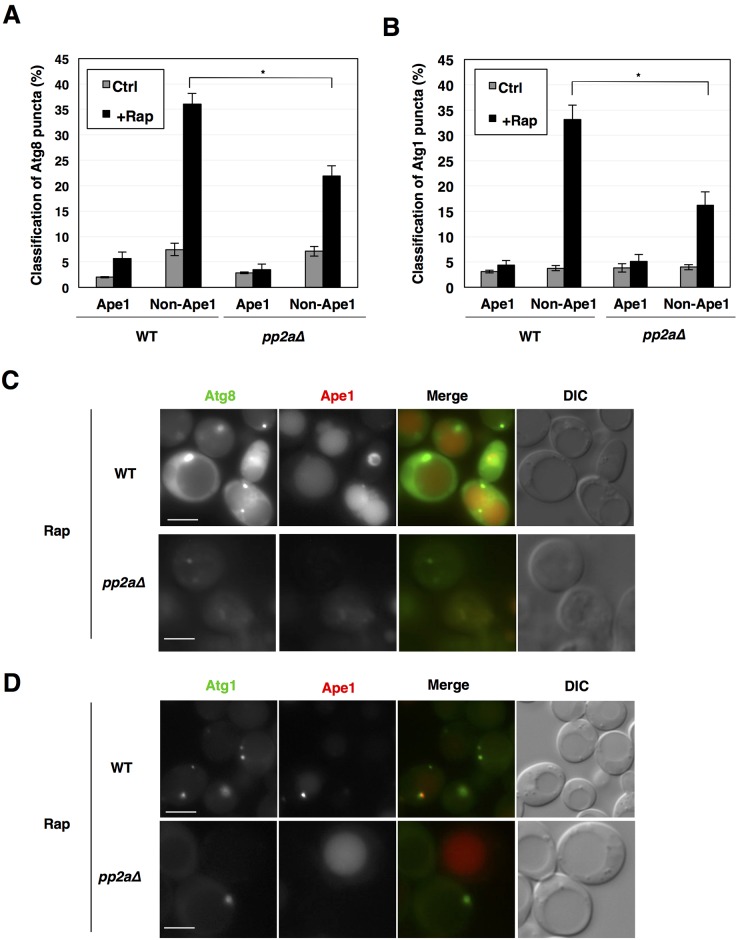
Characterization of rapamycin-induced Atg8 and Atg1 puncta. (A, B) Cells of strains SCU893 (wild type) and SCU2422 (*pph21*Δ *pph22*Δ) harboring plasmid pSCU1998 (pGFP-ATG8) or pSCU2138 (pATG1-GFP) in combination with pSCU2148 (pRFP-APE1) were treated with rapamycin for 1 h. GFP puncta that are colocalized and non-colocalized with RFP-Ape1 puncta were counted and are expressed as percentages. For examination of PAS formation, more than 100 cells with Atg8-marked puncta were counted and were scored. Microscope observations were performed at least twice independently to confirm reproducibility of the results. Data are shown as means ± errors. *, P < 0.01 (Fisher’s exact test). (C, D) Cell images with Atg8 and Atg1 puncta colocalized with or without Ape1 after rapamycin treatment are shown. Scale bars, 5 μm.

In wild-type cells, some Atg protein-marked PAS structures are colocalized with Ape1 puncta, which represent prApe1 oligomers both under growing and starvation conditions, whereas others are not [33, 34]. Ape1-colocalized and Ape1-noncolocalized PAS structures correspond to the Cvt pathway and others including nonselective autophagy, respectively. We observed colocalization of GFP-Atg8 or Atg1-GFP puncta with RFP-Ape1 puncta. Ape1-noncolocalized Atg8 and Atg1 puncta, but not Ape1-colocalized ones, were markedly increased in the wild-type cells after rapamycin treatment, whereas the increase was repressed in *pp2a*Δ cells (Fig 5A–5D). These findings indicated that PP2A is required for Ape1-independent PAS formation after TORC1 inactivation. This is consistent with the idea that PP2A is required for TORC1 inactivation-induced nonselective autophagy.

### Overexpression of non-phosphorylatable Atg13 recovers autophagy induction after rapamycin treatment in *pp2a*Δ cells

Overexpression of a non-phosphorylatable Atg13 mutant (Atg13-8SA) evokes autophagy induction in normal conditions, although it does not completely phenocopy TORC1 inactivation (rapamycin treatment): the autophagy induction was weaker than that in TORC1 inactive conditions [7]. This indicated that dephosphorylation of Atg13 is sufficient for autophagy induction to some extents. If deficient dephosphorylation of Atg13 is a main cause of the defect in autophagy induction in *pp2a*Δ cells, overexpression of Atg13-8SA in normal conditions might induce autophagy in the mutant cells, like in the wild-type cells. However, if *pp2a*Δ cells have additional critical defects in the mechanisms involved in autophagy induction, overexpression of Atg13-8SA could induce autophagy in the wild-type cells, but not in the *pp2a*Δ cells.

**Fig 6:**
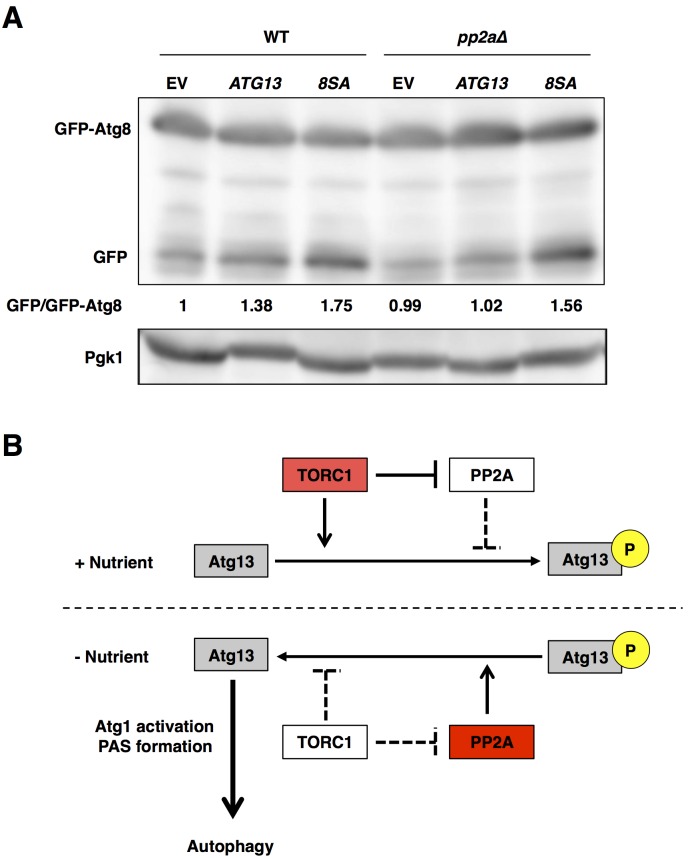
Overexpression of non-phosphorylatable Atg13 recovers autophagy induction after rapamycin treatment in *pp2a*Δ cells. (A) Cells of strains SCU893 (wild-type) and SCU4154 (*pph21*Δ *pph22*Δ) harboring plasmid an pSCU154 (empty vector; EV), pSCU1986 (pGAL1-ATG13; *ATG13*) or pSCU1987 (pGAL1-ATG13-8SA; *8SA*) in combination with pSCU1978 (pGFP-ATG8) cultured in raffinose-base media were added with 2% galactose for 3 h. Whole cell extracts were subjected to western blotting using an anti-GFP antibody. (B) In nutrient-rich conditions, TORC1 phosphorylates Atg13 and represses PP2A, promoting Atg13 phosphorylation. Whereas, in starvation conditions, TORC1 is inactivated, and activated PP2A mediates Atg13 dephosphorylation, promoting Atg13 dephosphorylation.

We overexpressed the wild-type and mutant *ATG13* under the control of *GAL* promoter. Autophagy was slightly induced in a galactose-based medium, as compared with a normal glucose-based medium (Fig 6A, EV and data not shown). Overexpression of wild-type Atg13 further evokes autophagy in the wild-type cells, but not in *pp2a*Δ cells (Fig 6A, *ATG13*). Overexpression of non-phosphorylatable Atg13 remarkably induced autophagy in the wild-type and *pp2a*Δ cells (Fig 6A, *8SA*). These findings supported the idea that deficient dephosphorylation of Atg13 is a critical cause of the defect in autophagy induction in PP2A-deleted cells.

## Discussion

### Autophagy induction by the orchestrated actions of TORC1 and PP2A

Here, we showed that PP2A-Cdc55 and PP2A-Rts1 are required for Atg13 dephosphorylation, Atg1 activation, PAS formation, and autophagy induction after the inactivation of TORC1. According to our results, we propose a model for the involvement of PP2A in autophagy induction (Fig 6B). In normal (nutrient-rich) conditions, TORC1 phosphorylates Atg13 and represses PP2A activity, inhibiting autophagy induction. Upon nutrient starvation and TORC1 inactivation, TORC1-mediated phosphorylation of Atg13 is repressed, and simultaneously PP2A is activated and promotes Atg13 dephosphorylation, which in turn promotes Atg1 activation and autophagy induction. Thus, the orchestrated actions of TORC1 and PP2A might be important to switch “on” and “off” autophagy rapidly and effectively. Note that Atg13 dephosphorylation still occurred in *pp2a*Δ cells after the inactivation of TORC1. These findings indicated that additional protein phosphatases involved in dephosphorylation of Atg13 might exist. It is likely that multiple phosphatases are cooperatively involved in Atg13 dephosphorylation and autophagy induction after the inactivation of TORC1.

Overexpression of the non-phosphorylatable Atg13-8SA induced autophagy in normal conditions and recovered autophagy flux in *pp2a*Δ and *cdc55*Δ *rts1*Δ cells [7] (this work). However, overexpression of *PPH21*, *PPH22*, *CDC55*, or *RTS1* did not evoke autophagy in normal conditions (data not shown). It is likely that dephosphorylation of Atg13 by overproduced PP2A did not overcome phosphorylation of them by TORC1 in these conditions. Alternatively, synergistic actions with other uncharacterized phosphatases might be required for autophagy induction.

### Other aspects of PP2A in autophagy induction

The simplest hypothesis is that PP2A directly dephosphorylates Atg13. All subunits of PP2A (Pph21, Pph22, Cdc55, Rts1, and Tpd3) were localized in the cytoplasm in addition to the nucleus in TORC1 active and inactive conditions (S7A–S7E Fig). Although we did not find accumulation of PP2A in the perivacuolar region as puncta after rapamycin treatment (S7A–S7E Fig), PP2A localized in the cytoplasm could interact with Atg13. Unfortunately, we could not detect a physical interaction between PP2A and Atg13, because interaction of PP2A with its substrates is generally too weak to detect using traditional methods, including yeast two-hybrid assay [35]. Atg13 dephosphorylation and autophagy induction after TORC1 inactivation were not suppressed by the loss of *CDC55* or *RTS1* individually, whereas it was impaired by the double deletion of *CDC55* and *RTS1*. Considering these results together, we concluded that PP2A-Cdc55 and PP2A-Rts1 have a redundant function in Atg13 dephosphorylation and autophagy induction after TORC1 inactivation. Atg13 has numerous phosphorylation sites and how multiple phosphorylation sites of Atg13 regulate Atg1 complex formation and autophagy induction is largely unknown [7, 36]. It is interesting to assess whether PP2A-Cdc55 and PP2A-Rts1 contribute to dephosphorylation of the same sites of Atg13 or different sites.

Several lines of assessment (GFP-Atg8 processing, Ape1 processing in *atg11*Δ, Rosella assay) all indicated that TORC1 inactivation-induced autophagy (nonselective autophagy) was compromised in *pp2a*Δ cells. Unexpectedly, the Pho8Δ60 assay [37, 38] showed no such reduction in autophagy flux in *pp2a*Δ cells (data not shown). These findings suggested that PP2A might affect Pho8 phosphatase activation in an autophagy-independent manner, leading to these conflicts. To test this idea, further analysis would be required in the future.

It was reported that the recruitment of Atg8 to the PAS after rapamycin treatment was increased in *atg1*Δ, *atg13*Δ, and *atg17*Δ mutants [33]. This was different from our present results using PP2A-deficient mutants, although the function of the Atg1 complex may be partially compromised in *pp2a*Δ cells. This conflict suggested that PP2A might also be required for other steps in TORC1 inactivation-induced PAS organization, in addition to Atg13 dephosphorylation.

### Does PP2A regulate autophagy positively or negatively?

In contrast to our conclusion, it was reported previously that PP2A inhibited autophagy, in that PP2A inactivation stimulated autophagy (monitored by GFP-Atg8 cleavage) under normal conditions [39]. To assess the roles of PP2A in autophagy induction, the previous study used a temperature-sensitive mutant strain, DEY217, harboring *pph21*Δ *pph22-172 pph3*Δ [40]. PP2A and PP4/Pph3 are not essential for viability in *S*. *cerevisiae*, although *pp2a*Δ cells showed growth defects [41]. PP2A and PP4/Pph3 redundantly control essential events, as the triple mutant *pph21*Δ *pph22*Δ *pph3*Δ is lethal [41]. However, PP4/Pph3 also has distinct functions from those of PP2A [42]. Therefore, this previous study that examined the effects on autophagy when the functions of PP2A and PP4 were both lost [39], which might lead to different results from ours. Alternatively, this conflict might result from an allele-specific mutation of *PPH22*. We believe that our study could provide a model for the involvement of PP2A in autophagy regulation in yeast. In other organisms, it has been reported that PP2A (or PP2A-related phosphatases) positively regulate autophagy induction, supporting our model (see below).

Aside from this, it is interesting how autophagy is induced in *pph21*Δ *pph22-172 pph3*Δ cells at restrictive temperatures. This mutant strain undergoes cell lysis at restrictive temperatures, which was suppressed by a high osmolarity [40]. These findings suggested that the mutant cells were defective in cell wall integrity and suffered from cell wall stress. Cell wall stress-activated MAPK Mpk1/Slt2 promotes mitophagy via phosphorylation of the mitochondrial outer-membrane protein Atg32 [43]. It is likely that the lack of PP2A and PP4 causes cell wall stress, which stimulates Mpk1 activation and mitophagy, promoting vacuolar degradation of Atg8. This is an intriguing possibility to be tested in the future.

### PP2A and autophagy in other organisms

Mammalian cells possess the ULK1 (Unc-51 like kinase-1) complex, which is homologous to the Atg1 complex, albeit with some dissimilarities. ULK1 (an Atg1 homolog) constitutively forms this complex with ATG13 and FIP200 (RB1CC1; Atg17 counterpart) under both nutrient-rich and nutrient-starved conditions [44, 45]. Mammalian TORC1 (mTORC1) phosphorylates not only ATG13 but also ULK1 [44–46], but phosphatases responsible for the dephosphorylation of ATG13 or ULK1 are currently ambiguous. Okadaic acid, an inhibitor of PP2A and PP2A-like phosphatases, represses autophagy [47, 48]. In addition, PP2A is required for starvation-induced autophagy in *Drosophila* cells [49]. Although the targets of PP2A are largely unknown in these organisms, it is likely that PP2A positively regulates Atg1/ULK1 complex function (even if not via complex formation) after the inactivation of TORC1 in other organisms, such as yeast. We predict that the regulation of Atg1 complex function and autophagy by the orchestrated actions of TORC1 and PP2A are widely conserved among eukaryotic cells.

Neurodegenerative diseases, such as Alzheimer's disease (AD), Parkinson's disease, Huntington's disease, and amyotrophic lateral sclerosis, share common cellular and molecular pathogenetic mechanisms involving aberrant misfolded proteins or peptide aggregation and deposition [50]. Autophagy degrades aggregated proteins and dysfunctional organelles. Mounting evidence has implicated defective autophagy in the pathogenesis of these neurodegenerative diseases, especially AD. Upregulation of autophagy can lead to decreased levels of these toxic aggregates, and is beneficial in the context of aging and various models of neurodegenerative diseases [51]. Chronic treatment of rapamycin induces autophagy, and blocks amyloid-β and the progression of AD-like cognitive deficits in an autophagy-dependent manner in the mouse model of AD [52, 53]. However, mTORC1 also controls diverse cellular events, and drugs targeting mTORC1 may cause undesirable side-effects, including immunosuppression. It may be better for therapeutic strategies for neurodegenerative diseases to target the Atg1 complex specifically.

Importantly, decrease in PP2A activity is a clinical feature of AD, although the molecular mechanism of the decrease remains elusive [54]. The blockage of PP2A by okadaic acid treatment or short hairpin RNA (shRNA)-mediated knockdown of PP2A catalytic subunit caused inhibition of basal autophagy and accumulation of ubiquitinated protein and protein aggregates in cultured neuronal cells [55]. These findings suggest a possibility that PP2A dysfunction compromised basal autophagy in the neuronal cells and causes AD [55]. Although how PP2A promotes autophagy in neuronal cells is unknown at present, it is tempting to speculate that PP2A promotes ULK1 complex function in these cells. PP2A-dificient yeast cells would be good tools to investigate the molecular basis of AD. To understand molecular mechanisms of PP2A-modulated autophagy flux should be important. The present study provides a novel insight into the molecular dissection of PP2A-regulated Atg1 complex function and a good model toward autophagy regulation in human cells.

## Supporting Information

S1 Fig PP2A is required for TORC1 inactivation-induced autophagy, but not the Cvt pathway. (A) Cells of strains SCU3720 (*atg11Δ*) and SCU3736 (*pph21Δ pph22Δ atg11Δ*) harboring plasmid pSCU1998 (pGFP-ATG8) were treated with rapamycin for 3 h. (B) Assessment of the Cvt pathway in PP2A-deficient cells. Cells of strains SCU893 (wild-type), SCU2422 (*pph21Δ pph22Δ*) and SCU4225 (*cdc55Δ rts1Δ*) were grown under normal (nutrient-rich) condition. Whole cell extracts were subjected to western blotting using an anti-Ape1 antibody.
(TIFF)

S2 Fig Quantification of autophagy flux using Rosella. (A) Cells of strains SCU893 (wild-type) and SCU2422 (*pph21Δ pph22Δ*) harboring plasmid pSCU2260 expressing Rosella (pH-sensitive GFP fused to RFP) were treated with rapamycin for 18 h (see Fig 1E). GFP and RFP signals in the cytoplasm and vacuole were examined using a microscope and image analysis software. Representative images of cells are shown. (B) Definitions of cytoplasmic GFP (GFPc) and RFP (RFPc) and vacuolar GFP (GFPv) and RFP (RFPv). For details, see “Materials and Methods”.
(TIFF)

S3 Fig PP2A-Cdc55 and PP2A-Rts1 are involved in nonselective autophagy induction. (A) Cell growth of *pp2aΔ and cdc55Δ rts1Δ* mutants. Cells of strains SCU893 (wild-type), SCU2422 (*pph21Δ pph22Δ*) and SCU4225 (*cdc55Δ rts1Δ*) were used. Serially 5-fold diluted cells of each strain were spotted from left to right on YPAD plates and incubated at 30°C for 1 day. (B) Cells of strains SCU3720 (*atg11Δ*) and SCU4069 (*cdc55Δ rts1Δ atg11Δ*) harboring plasmid pSCU1998 (pGFP-ATG8) were treated with rapamycin for 3 h. Whole cell extracts were subjected to western blotting using the anti-GFP antibody.
(TIFF)

S4 Fig PP2A is required for autophagy induction after nitrogen starvation. (A) The GFP-Atg8 cleavage assay indicates that autophagy induction after nitrogen starvation is compromised in pp2aΔ cells. Cells of strains SCU893 (wild-type) and SCU2422 (*pph21Δ pph22Δ*) harboring a plasmid pSCU1998 were transferred to SD-N medium and incubated for a further 3 h. (B) Cells of strains SCU3720 (*atg11Δ*) and SCU3736 (*pph21Δ pph22Δ atg11Δ*) were transferred to SD-N medium and incubated for a further 6 h. (C) Cells of strains SCU893 (wild-type) and SCU4225 (*cdc55Δ rts1Δ*) harboring plasmid pSCU1998 were transferred to SD-N medium and incubated for a further 3 h. (D) Cells of strains SCU3720 (*atg11Δ*) and SCU4069 (*cdc55Δ rts1Δ atg11Δ*) were transferred to SD-N medium and incubated for a further 6 h.
(TIFF)

S5 Fig PP2A is required for pre-autophagosomal structure localization of Atg8 after the activation of TORC1. (A, B) Cells of strains SCU893 (wild-type) and SCU2422 (*pph21Δ pph22Δ*) harboring plasmid pSCU1998 (pGFP-ATG8) were treated with rapamycin for 1 h. Scale bars, 5 μm. Cells with GFP puncta were counted and are expressed as percentages in (B). (C, D) Cells of strains SCU893 (wild-type) and SCU3174 (*pph21Δ pph22Δ*) harboring plasmid pSCU1960 (pATG1-GFP) were treated with rapamycin for 1 h. Cells with GFP puncta were counted and are expressed as percentages in (D). For examination of PAS formation, more than 100 cells with Atg8- or Atg1-marked puncta were counted and were scored. Microscope observations were performed at least twice independently to confirm reproducibility of the results. Data are shown as means ± errors. *, P < 0.01 (Fisher’s exact test).
(TIFF)

S6 Fig PP2A-Cdc55 and PP2A-Rts1 are involved in PAS localization of Atg8 after the activation of TORC1. (A, B) Cells of strains SCU893 (wild-type) and SCU4225 (*cdc55Δ rts1Δ*) harboring plasmid pSCU1998 (pGFP-ATG8) were treated with rapamycin for 1 h. Scale bars, 5 μm. Cells with GFP puncta were counted and are expressed as percentages in (B). For examination of PAS formation, more than 100 cells with Atg8-marked puncta were counted and were scored. Microscope observations were performed at least twice independently to confirm reproducibility of the results. Data are shown as means ± errors. *, P < 0.01 (Fisher’s exact test).
(TIFF)

S7 Fig Localization of PP2A under normal and starvation conditions. Cells of strains SCU1575 (*PPH21-GFP*), SCU1576 (*PPH22-GFP*), SCU1419 (*CDC55-GFP*), SCU1598 (*RTS1-GFP*) and SCU1653 (*TPD3-GFP*) were treated with rapamycin for 1 h. Representative GFP images are shown in each strain. Scale bars, 5 μm.
(TIFF)

S1 Table Yeast strains used in this study. (DOCX)

S2 Table Plasmids used in this study. (DOCX)

## Abbreviations

AD: Alzheimer's disease
ATG: autophagy-related
CDK: cyclin-dependent kinase
Cvt: cytoplasm to vacuole targeting
GFP: green fluorescent protein
PAS: pre-autophagosomal structure
PMSF: phenylmethylsulfonylfluoride
PP2A: protein phosphatase 2A
PP4: protein phosphatase 4
PP6: protein phosphatase 6
RFP: red fluorescent protein
TORC1: target of rapamycin complex 1
ULK1: Unc-51 like kinase-1
