# Supplementary figures and images for: Orchestrated Action of PP2A Antagonizes Atg13 Phosphorylation and Promotes Autophagy after the Inactivation of TORC1
Source: PLoS One. 2016 Dec 14;11(12):e0166636. doi: 10.1371/journal.pone.0166636 (PMC5156417; doi:10.1371/journal.pone.0166636)

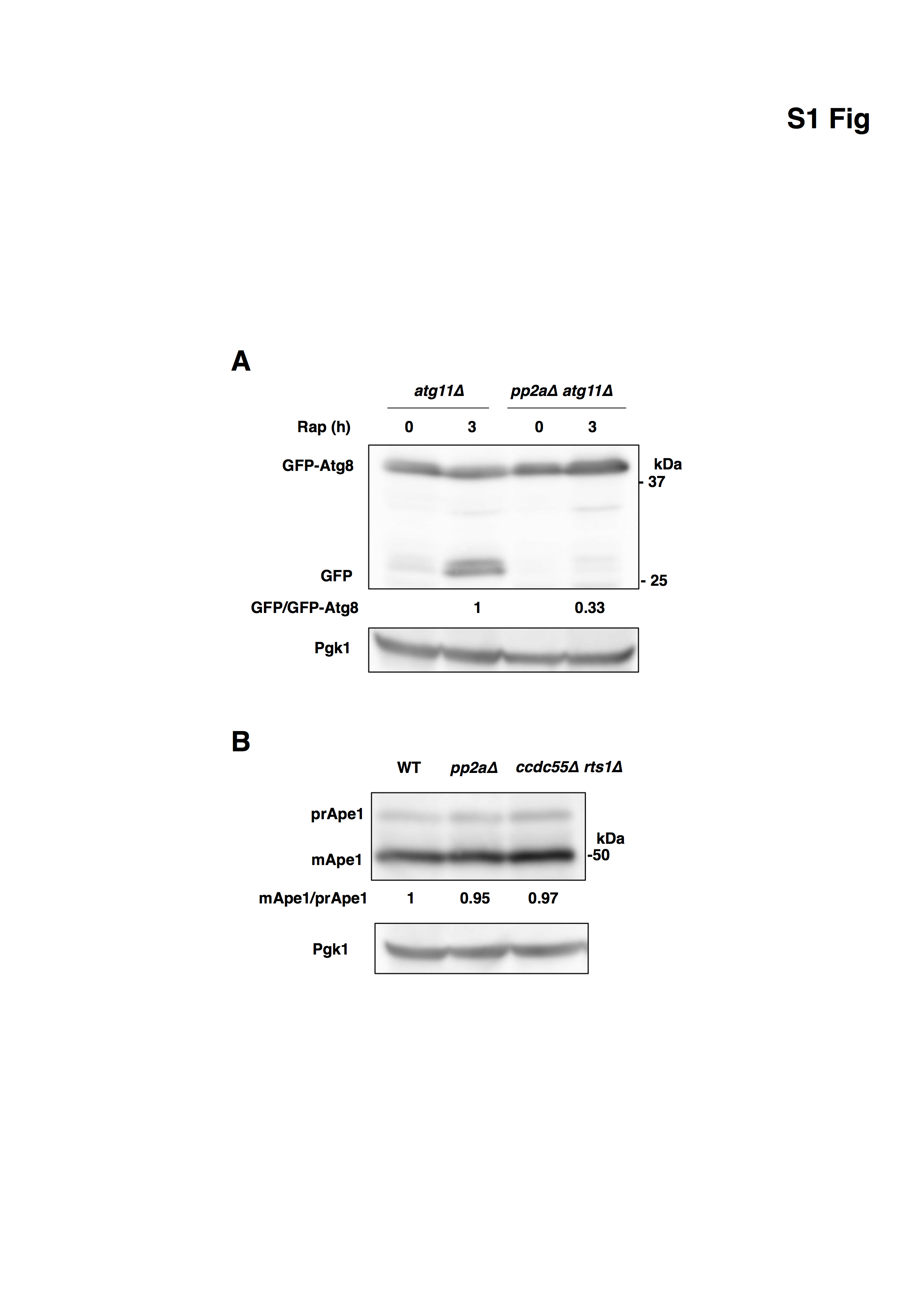

Supplement: S1 Fig — (A) Cells of strains SCU3720 (atg11Δ) and SCU3736 (pph21Δ pph22Δ atg11Δ) harboring plasmid pSCU1998 (pGFP-ATG8) were treated with rapamycin for 3 h. (B) Assessment of the Cvt pathway in PP2A-deficient cells. Cells of strains SCU893 (wild-type), SCU2422 (pph21Δ pph22Δ) and SCU4225 (cdc55Δ rts1Δ) were grown under normal (nutrient-rich) condition. Whole cell extracts were subjected to western blotting using an anti-Ape1 antibody. (TIFF) [file pone.0166636.s001.tiff]

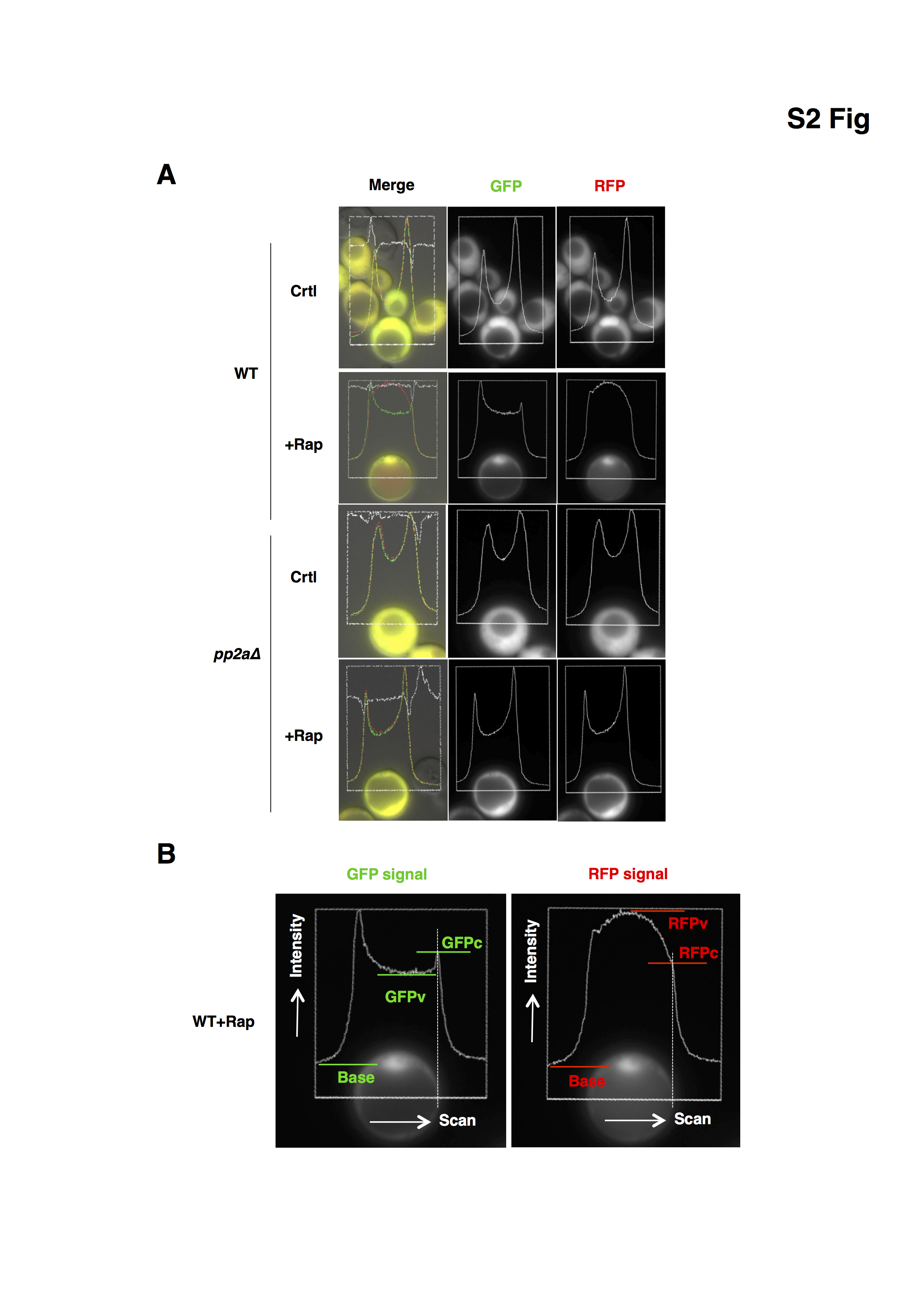

Supplement: S2 Fig — (A) Cells of strains SCU893 (wild-type) and SCU2422 (pph21Δ pph22Δ) harboring plasmid pSCU2260 expressing Rosella (pH-sensitive GFP fused to RFP) were treated with rapamycin for 18 h (see Fig 1E). GFP and RFP signals in the cytoplasm and vacuole were examined using a microscope and image analysis software. Representative images of cells are shown. (B) Definitions of cytoplasmic GFP (GFPc) and RFP (RFPc) and vacuolar GFP (GFPv) and RFP (RFPv). For details, see “Materials and Methods”. (TIFF) [file pone.0166636.s002.tiff]

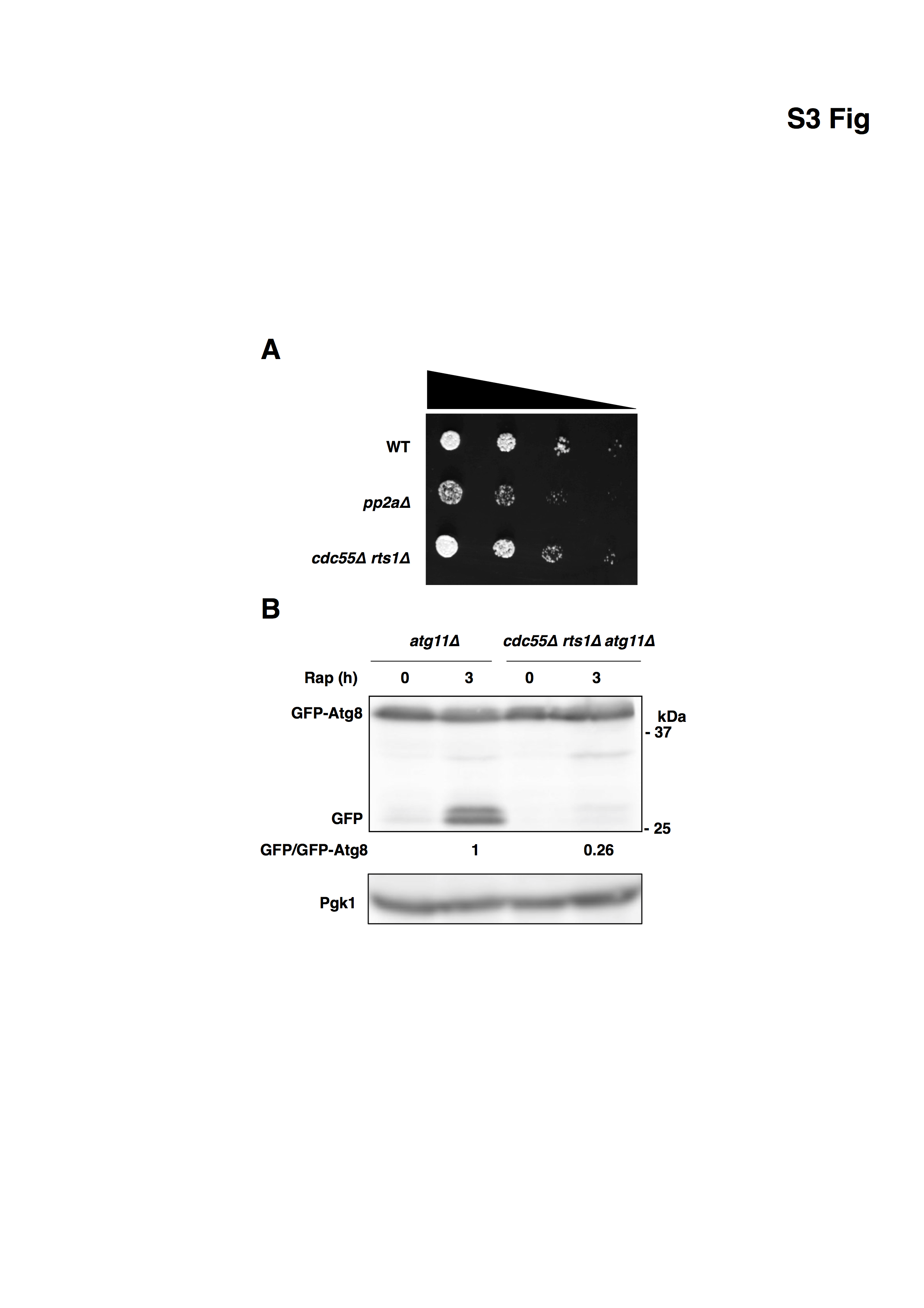

Supplement: S3 Fig — (A) Cell growth of pp2aΔ and cdc55Δ rts1Δ mutants. Cells of strains SCU893 (wild-type), SCU2422 (pph21Δ pph22Δ) and SCU4225 (cdc55Δ rts1Δ) were used. Serially 5-fold diluted cells of each strain were spotted from left to right on YPAD plates and incubated at 30°C for 1 day. (B) Cells of strains SCU3720 (atg11Δ) and SCU4069 (cdc55Δ rts1Δ atg11Δ) harboring plasmid pSCU1998 (pGFP-ATG8) were treated with rapamycin for 3 h. Whole cell extracts were subjected to western blotting using the anti-GFP antibody. (TIFF) [file pone.0166636.s003.tiff]

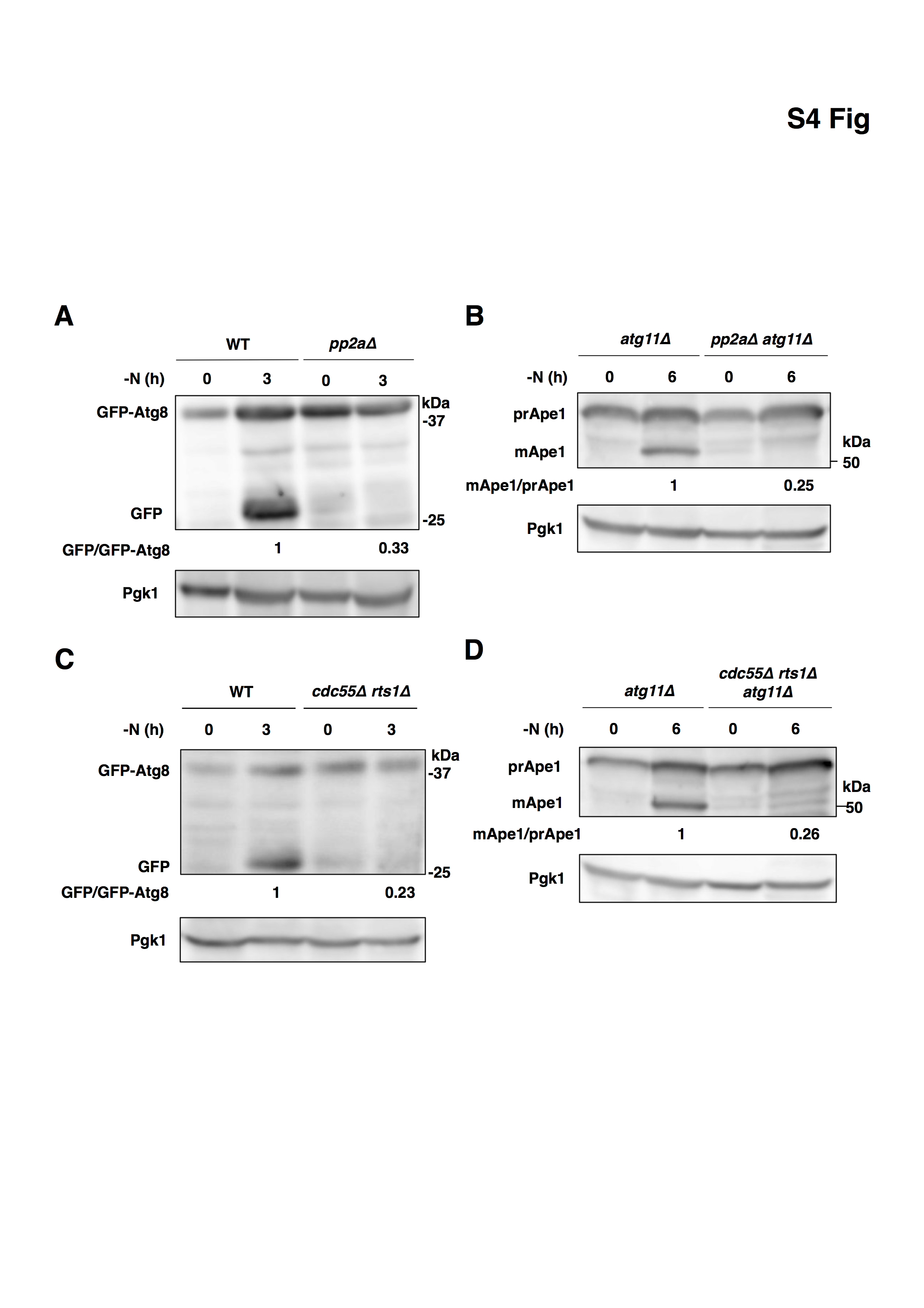

Supplement: S4 Fig — (A) The GFP-Atg8 cleavage assay indicates that autophagy induction after nitrogen starvation is compromised in pp2aΔ cells. Cells of strains SCU893 (wild-type) and SCU2422 (pph21Δ pph22Δ) harboring a plasmid pSCU1998 were transferred to SD-N medium and incubated for a further 3 h. (B) Cells of strains SCU3720 (atg11Δ) and SCU3736 (pph21Δ pph22Δ atg11Δ) were transferred to SD-N medium and incubated for a further 6 h. (C) Cells of strains SCU893 (wild-type) and SCU4225 (cdc55Δ rts1Δ) harboring plasmid pSCU1998 were transferred to SD-N medium and incubated for a further 3 h. (D) Cells of strains SCU3720 (atg11Δ) and SCU4069 (cdc55Δ rts1Δ atg11Δ) were transferred to SD-N medium and incubated for a further 6 h. (TIFF) [file pone.0166636.s004.tiff]

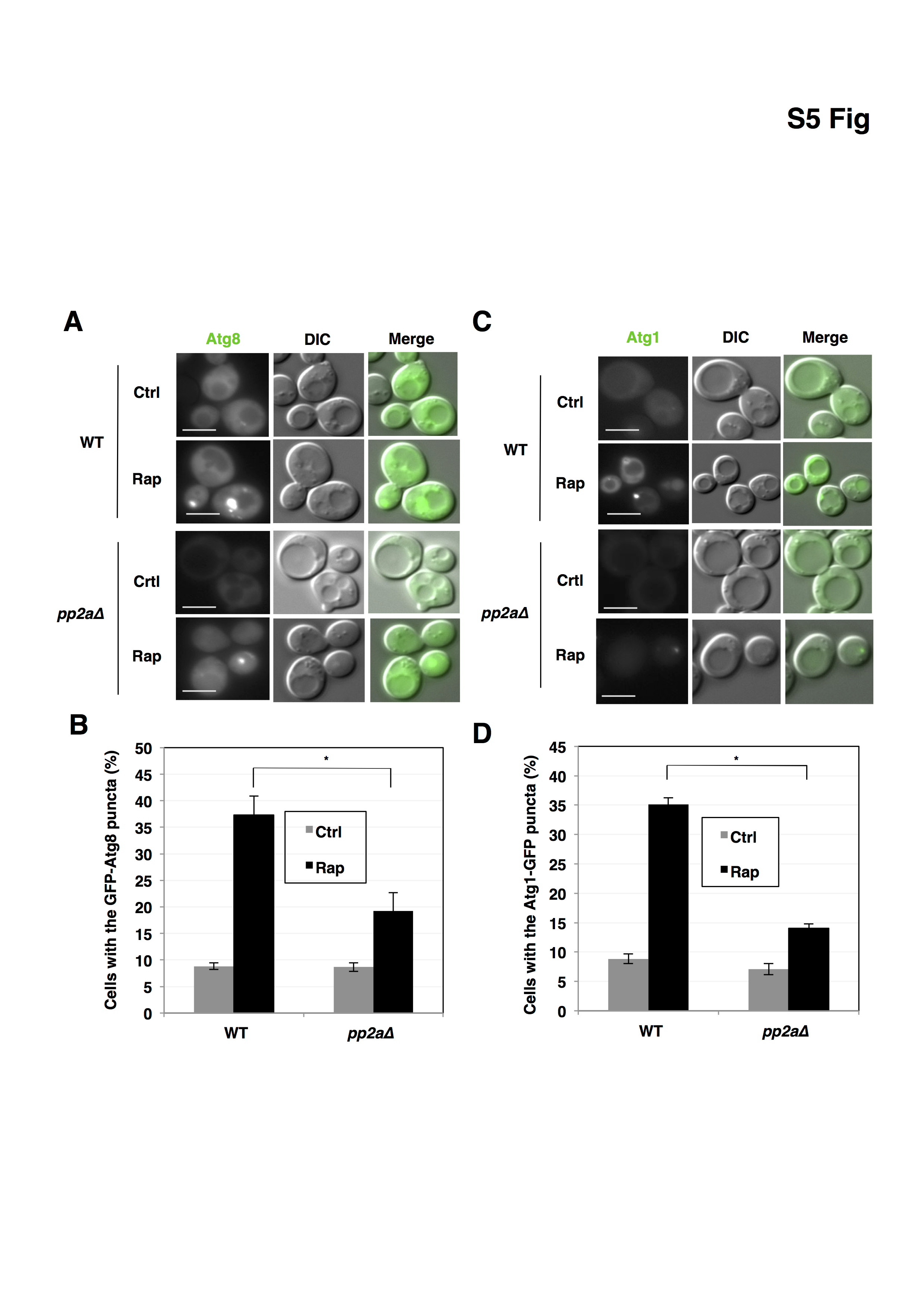

Supplement: S5 Fig — (A, B) Cells of strains SCU893 (wild-type) and SCU2422 (pph21Δ pph22Δ) harboring plasmid pSCU1998 (pGFP-ATG8) were treated with rapamycin for 1 h. Scale bars, 5 μm. Cells with GFP puncta were counted and are expressed as percentages in (B). (C, D) Cells of strains SCU893 (wild-type) and SCU3174 (pph21Δ pph22Δ) harboring plasmid pSCU1960 (pATG1-GFP) were treated with rapamycin for 1 h. Cells with GFP puncta were counted and are expressed as percentages in (D). For examination of PAS formation, more than 100 cells with Atg8- or Atg1-marked puncta were counted and were scored. Microscope observations were performed at least twice independently to confirm reproducibility of the results. Data are shown as means ± errors. *, P < 0.01 (Fisher’s exact test). (TIFF) [file pone.0166636.s005.tiff]

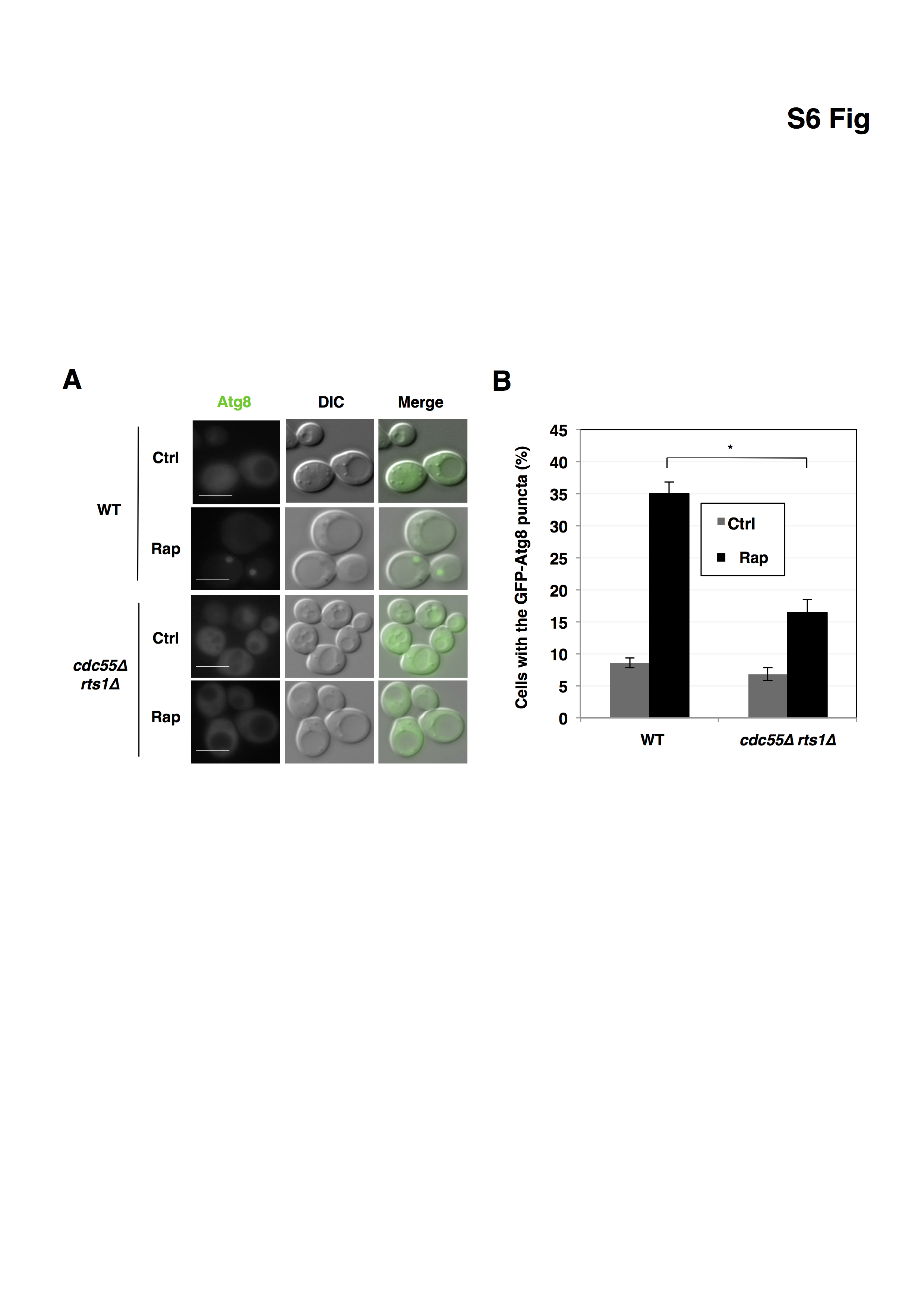

Supplement: S6 Fig — (A, B) Cells of strains SCU893 (wild-type) and SCU4225 (cdc55Δ rts1Δ) harboring plasmid pSCU1998 (pGFP-ATG8) were treated with rapamycin for 1 h. Scale bars, 5 μm. Cells with GFP puncta were counted and are expressed as percentages in (B). For examination of PAS formation, more than 100 cells with Atg8-marked puncta were counted and were scored. Microscope observations were performed at least twice independently to confirm reproducibility of the results. Data are shown as means ± errors. *, P < 0.01 (Fisher’s exact test). (TIFF) [file pone.0166636.s006.tiff]

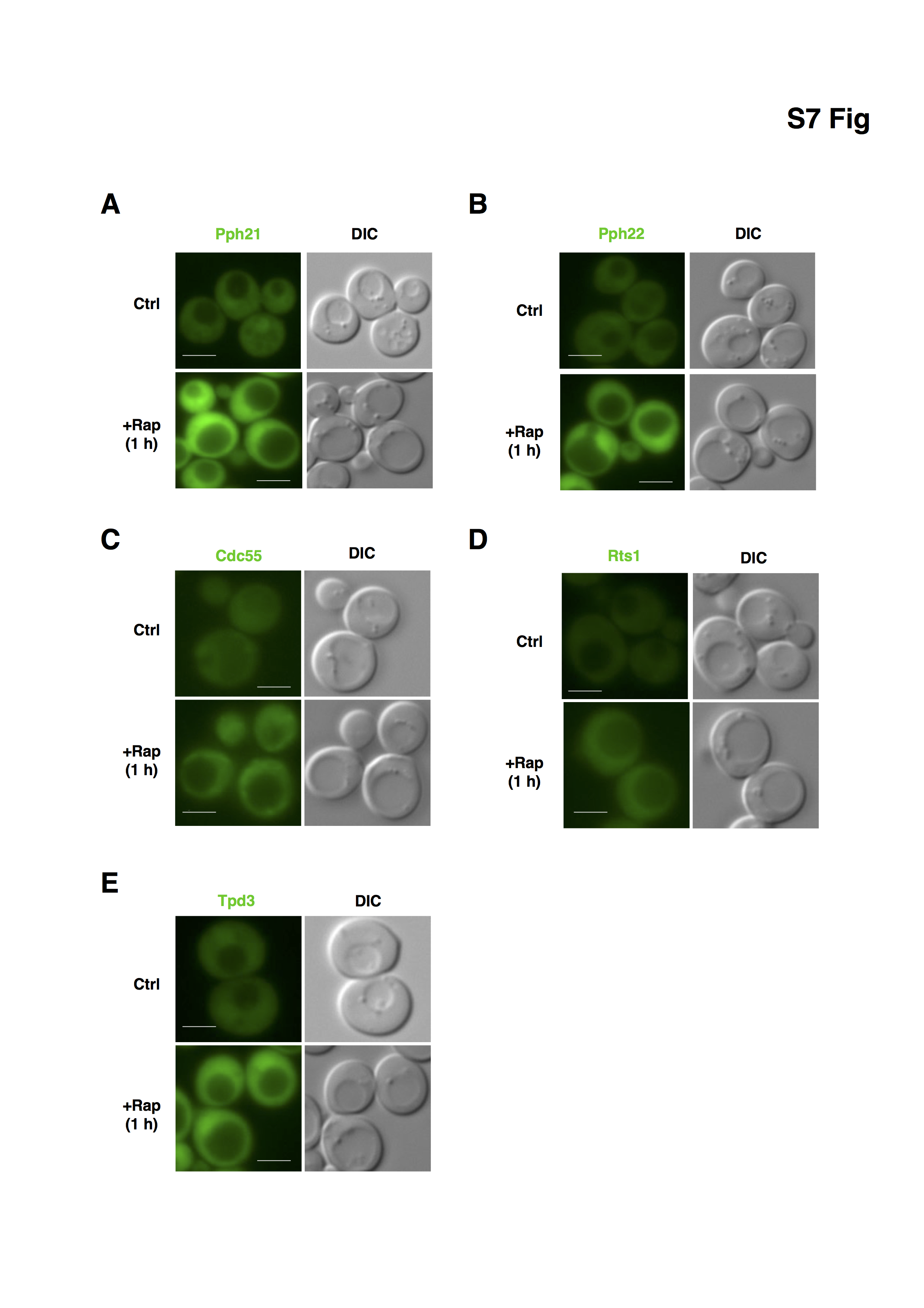

Supplement: S7 Fig — Cells of strains SCU1575 (PPH21-GFP), SCU1576 (PPH22-GFP), SCU1419 (CDC55-GFP), SCU1598 (RTS1-GFP) and SCU1653 (TPD3-GFP) were treated with rapamycin for 1 h. Representative GFP images are shown in each strain. Scale bars, 5 μm. (TIFF) [file pone.0166636.s007.tiff]
