# Supplementary material for: Orchestrated Action of PP2A Antagonizes Atg13 Phosphorylation and Promotes Autophagy after the Inactivation of TORC1
Source: PLoS One. 2016 Dec 14;11(12):e0166636. doi: 10.1371/journal.pone.0166636 (PMC5156417; doi:10.1371/journal.pone.0166636)
Supplement: S1 Table — (DOCX) [file pone.0166636.s008.docx]

**S1 Table. Yeast strains used in this study**

| Name | Description (source) |
| --- | --- |
| W303a | *Mata ura3 his3 leu2 trp1 ade2 can1* (lab stock) |
| BY4741 | *Mata leu2∆ ura3∆0 his3∆1 met15∆0* (lab stock) |
| BJ2168 | *MATa leu2 trp1 ura3-52 prb1-1122 prc1-407 pep4-3* (Y. Kamada) |
| SCU893 | W303a *bar1::hisG* (lab stock) |
| SCU1419 | BY4741 *CDC55-GFP* ([Huh et al., 2003](#_ENREF_13)) |
| SCU1575 | BY4741 *PPH21-GFP* ([Huh et al., 2003](#_ENREF_13)) |
| SCU1576 | BY4741 *PPH22-GFP* ([Huh et al., 2003](#_ENREF_13)) |
| SCU1598 | BY4741 *RTS1-GFP* ([Huh et al., 2003](#_ENREF_13)) |
| SCU1653 | BY4741 *TPD3-GFP* ([Huh et al., 2003](#_ENREF_13)) |
| SCU2142 | BY4741 *sit4::kanMX* ([Winzeler et al., 1999](#_ENREF_53)) |
| SCU2422 | W303a *pph21::CgTRP1* *pph22::CgHIS3* (NBRP) ([Sakumoto et al., 2002](#_ENREF_36)) |
| SCU3086 | BY4741 *pph21::kanMX* ([Winzeler et al., 1999](#_ENREF_53)) |
| SCU3087 | BY4741 *pph22::kanMX* ([Winzeler et al., 1999](#_ENREF_53)) |
| SCU3088 | BY4741 *pph3::kanMX* ([Winzeler et al., 1999](#_ENREF_53)) |
| SCU3720 | W303a *bar1::hisG* *atg11::kanMX* (this study) |
| SCU3736 | W303a *pph21::CgTRP1* *pph22::CgHIS3 atg11::kanMX* (this study) |
| SCU4069 | W303a *cdc55::hphMX* *rts1::kanMX atg11::natMX* (this study) |
| SCU4154 | W303a *pph21::CgTRP1 pph22::kanMX* (this study) |
| SCU4221 | W303a *bar1::hisG* *cdc55::hphMX* (this study) |
| SCU4223 | W303a *bar1::hisG* *rts1::kanMX* (this study) |
| SCU4225 | W303a *bar1::hisG* *cdc55::hphMX rts1::kanMX* (this study) |
| SCU4231 | W303a *bar1::hisG* *ATG13-GFP::his3MX* (this study) |
| SCU4233 | W303a *pph21::CgTRP1 pph22::kanMX ATG13-GFP::his3MX* (this study) |
| SCU4137 | W303a *bar1::hisG* *ATG17-GFP::his3MX* (this study) |
| SCU4139 | W303a *pph21::hphMX pph22::kanMX ATG17-GFP::his3MX* (this study) |
| SCU4625 | W303a *pph21::hphMX pph22::kanMX* (this study) |
