# Supplementary material for: Orchestrated Action of PP2A Antagonizes Atg13 Phosphorylation and Promotes Autophagy after the Inactivation of TORC1
Source: PLoS One. 2016 Dec 14;11(12):e0166636. doi: 10.1371/journal.pone.0166636 (PMC5156417; doi:10.1371/journal.pone.0166636)
Supplement: S2 Table — (DOCX) [file pone.0166636.s009.docx]

**S2 Table. Plasmids used in this study**

| Name | Description (source) |
| --- | --- |
| pSCU134 | [pRS416GAL1] *URA3 CEN* ([Christianson et al., 1992](#_ENREF_5)) |
| pSCU452 | pFA6-hphMX4 (PCR template) ([Goldstein and McCusker, 1999](#_ENREF_9)) |
| pSCU1960 | [pRS414] *ATG1-GFP TRP1 CEN* (this study) |
| pSCU1978 | [pRS315] *GFP-ATG8 LEU2 CEN* (this study) |
| pSCU1984 | [YEp352] *ATG13 URA3 2µ* ([Kamada et al., 2010](#_ENREF_20)) |
| pSCU1986 | [p416GAL1] *ATG13 2µ URA3* ([Kamada et al., 2010](#_ENREF_20)) |
| pSCU1987 | [p416GAL1] *ATG13-8SA 2µ URA3* ([Kamada et al., 2010](#_ENREF_20)) |
| pSCU1998 | [pRS316] *GFP-ATG8 URA3 CEN* ([Suzuki et al., 2001](#_ENREF_46)) |
| pSCU2138 | [pRS316] *ATG1-GFP URA3 CEN3* ([Kondo-Okamoto et al., 2012](#_ENREF_23)) |
| pSCU2140 | [pRS414] *mRFP-APE1 TRP1 CEN* ([Shintani and Reggiori, 2008](#_ENREF_41)) |
| pSCU2254 | [pRS316] *PGK1-GFP URA3 CEN* ([Welter et al., 2014](#_ENREF_52)) |
| pSCU2260 | [YEplac181] *Rosella* *2µ LEU2* ([Rosado et al., 2008](#_ENREF_35)) |
